# Halogen-Doped Chevrel Phase Janus Monolayers for Photocatalytic Water Splitting

**DOI:** 10.3390/nano13020368

**Published:** 2023-01-16

**Authors:** Ekaterina V. Sukhanova, Nursultan E. Sagatov, Aleksandr S. Oreshonkov, Pavel N. Gavryushkin, Zakhar I. Popov

**Affiliations:** 1 Laboratory of Acoustic Microscopy, Emanuel Institute of Biochemical Physics of Russian Academy of Sciences, 119334 Moscow, Russia; 2 Laboratory of Phase Transformations and State Diagrams of the Earth’s Matter at High Pressures, Sobolev Institute of Geology and Mineralogy, Siberian Branch of Russian Academy of Sciences, 630090 Novosibirsk, Russia; 3 Laboratory of Molecular Spectroscopy, Kirensky Institute of Physics, Federal Research Center KSC SB RAS, 660036 Krasnoyarsk, Russia; 4 School of Engineering and Construction, Siberian Federal University, 660041 Krasnoyarsk, Russia; 5 Geology Geophysics Department, Novosibirsk State University, 630090 Novosibirsk, Russia

**Keywords:** TMDs, non-van der Waals monolayers, Mo6S8, Mo3S4, 2D materials, exfoliation, OER, HER, nanomaterials

## Abstract

Chevrel non-van der Waals crystals are promising candidates for the fabrication of novel 2D materials due to their versatile crystal structure formed by covalently bonded (Mo_6_X_8_) clusters (X–chalcogen atom). Here, we present a comprehensive theoretical study of the stability and properties of Mo-based Janus 2D structures with Chevrel structures consisting of chalcogen and halogen atoms via density functional theory calculations. Based on the analysis performed, we determined that the S_2_Mo_3_I_2_ monolayer is the most promising structure for overall photocatalytic water-splitting application due to its appropriate band alignment and its ability to absorb visible light. The modulated Raman spectra for the representative structures can serve as a blueprint for future experimental verification of the proposed structures.

## 1. Introduction

For the most part, the investigation of two-dimensional structures is focused on the materials obtained from layered crystals such as graphite, hexagonal boron nitride (h-BN), or transition metal dichalcogenides (TMDs). The main feature of these crystals is a weak van der Waals interaction between the layers, making it possible to easily cleave them into monolayers [1]. Reducing the dimension of the material leads to the appearance of unique new properties. For example, 2D nanomaterials are usually characterized by higher values of carrier mobility and conductivity in comparison with bulk crystals due to changes in electronic properties caused by the quantum confinement effect. Therefore, the search, investigation, and fabrication of new 2D nanomaterials from non-van der Waals crystals has attracted enormous attention [2]. Bulk materials, such as iron pyrite [3] and α-germanium [3], have relatively weak covalent bonds and can be exfoliated. Moreover, the achievements in liquid-phase exfoliation techniques have led to the rapid development of a second approach to 2D materials’ fabrication, which is the cleaving of non-van der Waals bulk materials into individual monolayers [2]. The successful application of this approach has led to the expansion of the 2D nanomaterials family and the fabrication of stable and easily processable nanomaterials [2].

In the 2D form, the TMDs are widely represented by MX_2_ stoichiometry, where M = Mo, V, W and X = S, Se, Te. In this stoichiometry, multiple phases have been experimentally observed—the hexagonal H phase and the tetragonal T and T’ (or Td) phases, which exhibited completely different electronic properties: in the case of MoS_2_, the H phase is a semiconductor, while the T phases are metals. Furthermore, several metastable TMD phases have been theoretically proposed, among which are the square-octagonal (S) and disordered square-octagonal (S’) monolayers [4], another hexagonal H’ phase [5], and a triclinic-structured A’ phase [6,7]. Moreover, several non-stoichiometric monolayers in the M-X system have been proposed [8,9,10,11] and some of them successfully fabricated [12,13]. The above indicate the prospects of the search for and investigation of new monolayer structures in the M-X systems that can be produced from non-van der Waals crystals.

The ternary molybdenum chalcogenides, or the Chevrel phases [14], are the non-van der Waals crystals of molybdenum chalcogenides, exhibiting compelling properties for next-generation battery materials, electrocatalysts, and other energy applications [15]. In general, Chevrel phases have a formula of M*_x_*Mo_6_X_8_ where X is a chalcogen atom (S, Se, Te) and M is a metal that can have a different valency (*x* varying from 0 to 4) [16]. Chevrel phases demonstrate the potential for application in battery materials [17,18,19,20], catalysts [21], thermoelectric materials [22], and superconductivity [23,24]. Binary compounds formed only by molybdenum clusters Mo_6_X_8_ are also interesting. In such compounds, Mo_6_ octahedra are surrounded by X_8_ cubes in which X atoms are located above the center of each Mo face, and such clusters are interconnected by Mo–X interactions, forming an ordered structure. Most Mo_6_X_8_ crystals have rhombohedral symmetry and are characterized by an $R\bar{3}$ space group [16]. A facile approach to synthesizing large-scale and high-purity Mo_6_S_8_ nanosheets by applying an iodine vapor transport reaction was proposed [25]. Moreover, in Chevrel phases, chalcogen atoms can be partially substituted by halogen atoms [26,27,28,29] or other chalcogen atoms [30], leading to the formation of X_a_Mo_6_Y_8-a_ structures, for example Mo_6_S_6_Br_2_ [28] or Mo_6_S_6.8_Te_1.2_ [30].

Recently, Janus monolayers [31,32,33,34], whose main feature is the presence of two inequivalent surfaces, have attracted significant attention due to their possible application in the photocatalytic water-splitting process [35,36,37,38], leading to the production of environmentally friendly hydrogen fuel [39,40]. The TMDs also include Janus-type monolayers [41,42], which can be obtained from the structures by replacing the chalcogen atoms of one surface with chalcogen atoms of another type [6,43,44,45,46]. Some of the proposed structures were recently successfully fabricated [41,42,47,48], resulting in intensive research into new possible Janus structures [6].

In this work, for the first time, we performed a comprehensive theoretical investigation of the stability and electronic and optical properties of novel Mo-based Janus Chevrel monolayers X_4_Mo_6_Y_4_ (X ≠ Y = O, S, Se, Te and X = O, S, Se, Te; Y = F. Cl, Br, I) and investigated the possibility of their application in photocatalytic water splitting.

## 2. Materials and Methods

Structure relaxation and calculations of electronic and optical properties based on density functional theory (DFT) [49,50] were carried out by the VASP program package [51,52,53]. The exchange-correlation functional was calculated via generalized gradient approximation (GGA) within Perdew–Burke–Ernzerhof (PBE) parameterization [54]. The projector-augmented wave (PAW) [55] basis set technique was used. The energy cutoff of plane waves were set to be equal to 520 eV. The first Brillouin zone was sampled according to the Monkhorst–Pack scheme [56] with a k-point mesh of 8 × 8 × 1. Atomic structure relaxation was carried out until the energy variation between the two steps of the electronic loop became less than 10^−8^ eV and there was less than10^−7^ eV between ionic steps. A vacuum region of at least 15 Å was chosen to avoid an artificial interaction between structures in non-periodic directions. Phonon calculations were carried out using the PHONOPY program package [57]. Charge redistribution in all considered systems was studied using Bader charge analysis [58,59]. For the atomic structure visualization, we used VESTA software [60] (version 3.4.5).

Modeling of Raman spectra was performed within the framework of DFT [61], using GGA [62] with an exchange-correlation PBE functional [54], implemented in CASTEP code [63]. Norm-conserving pseudopotentials were also used. The plane-wave cutoff was set to 740 eV and the k-point spacing was less than 0.05 Å^−1^ for all studied compounds. Monolayer structures were relaxed until the maximum forces became less than 0.03 eV/Å and the maximum stress was smaller than 0.05 GPa. Meanwhile, in the case of bulk Mo_6_S_8_, the same criteria were chosen as 0.01 eV/Å and 0.02 GPa. The tolerance for finding ground-state wavefunctions was set to 1 × 10^−8^ eV. Density functional perturbation theory was used for the calculation of phonon spectra at the center of the Brillouin zone and was also used for the calculation of Raman tensor components (finite displacement method [64,65]).

## 3. Results

**Figure 1:**
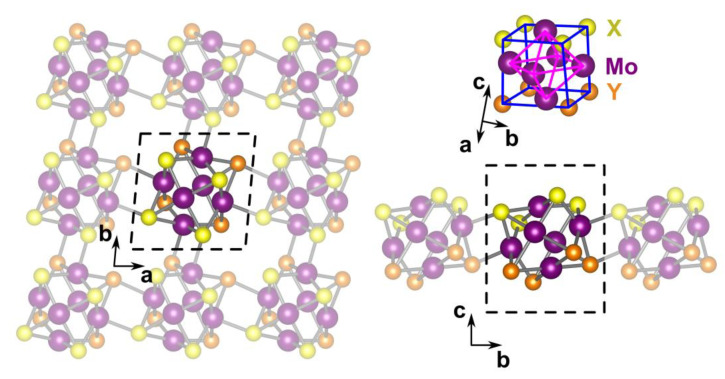
Atomic structure of a monolayer X_4_Mo_6_Y_4_ Chevrel phase (X/Y = O, S, Se, Te or X = O, S, Se, Te; Y = F. Cl, Br, I) and a single X_4_Mo_6_X_4_ cluster. The unit cell is highlighted by a black dashed line. Mo_6_ octahedra and X_4_Y_4_ cubes are marked by purple and blue lines, respectively.

The unit cell of Chevrel monolayers contains 18 Mo, 12 X, and 12 Y atoms (X = O, S, Se, Te; Y = O, S, Se, Te, F, Cl, Br, I) and consists of a single X_4_Mo_6_Y_4_ cluster located at some angle to the *c* axis (see Figure 1). The cluster connects with each of the four neighboring clusters by one Mo–X and one Mo–Y bond. The optimized cell parameters for each considered chemical composition are presented in Table 1 and Table 2.

Chevrel monolayers can be potentially fabricated by the cleavage of corresponding bulk ternary molybdenum chalcogenides along the (110) direction for non-Janus compounds. To consider this possibility, the exfoliation energy ($E_{exf}$) was estimated through the equation proposed in [66]:

$$E_{exf}=\frac{E_{ML}-E_{bulk}/m}{A}$$

where $E_{ML}$ is the total energy of a monolayer, $E_{bulk}$ is the total energy of bulk material consisting of m monolayers (in our case m=1), and A is the in-plane area according to the relaxed bulk unit cell. For the Mo_3_S_4_ monolayer, $E_{exf}$ is equal to 79.54 meV/Å^2^, for the Mo_3_Se_4_ monolayer it is 67.90 meV/Å^2^, and for the Mo_3_Te_4_ monolayer it is 60.80 meV/Å^2^. As expected, the obtained values exceed the characteristic values for Van der Waals layered crystals such as graphite (17–25 meV/Å^2^) or TMDs (~50 meV/Å^2^); however, the value is comparable with the estimations of the exfoliation energy for non-Van der Waals crystals [67,68,69] and even less than the value for experimentally exfoliated hematene [70]. Later, we divided the consideration of X_4_Mo_6_Y_4_ Chevrel phases (X/Y = O, S, Se, Te or X = O, S, Se, Te; Y = F. Cl, Br, I) into two subclasses: Ch^1^_2_Mo_3_Ch^2^_2_ (Ch^1^/Ch^2^ = O, S, Se, Te) and Ch_2_Mo_3_Hal_2_ (Ch = O, S, Se, Te and Hal = F, Cl, Br, I) monolayers.

**Figure 2:**
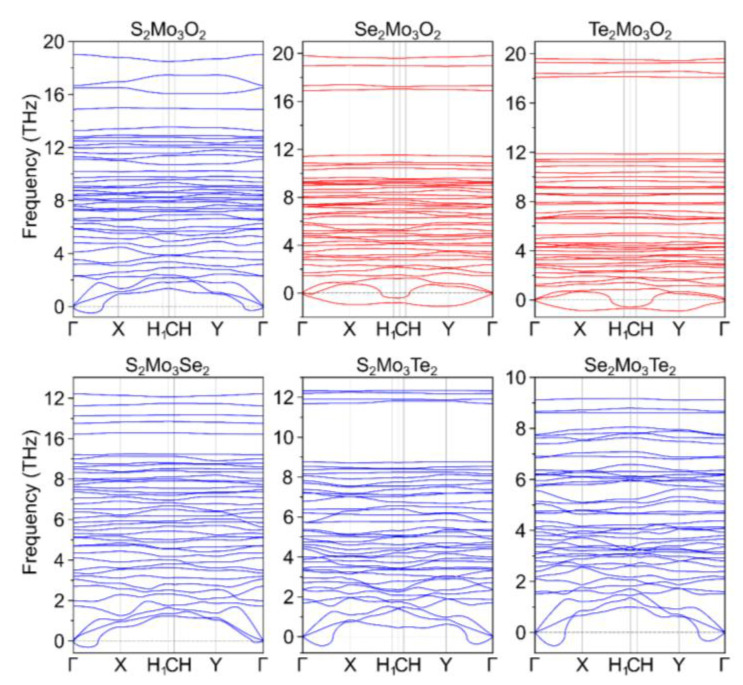
Phonon dispersion spectra for Ch^1^_2_Mo_3_Ch^2^_2_ (Ch^1^/Ch^2^ = O, S, Se, Te) Chevrel phases.

**Figure 3:**
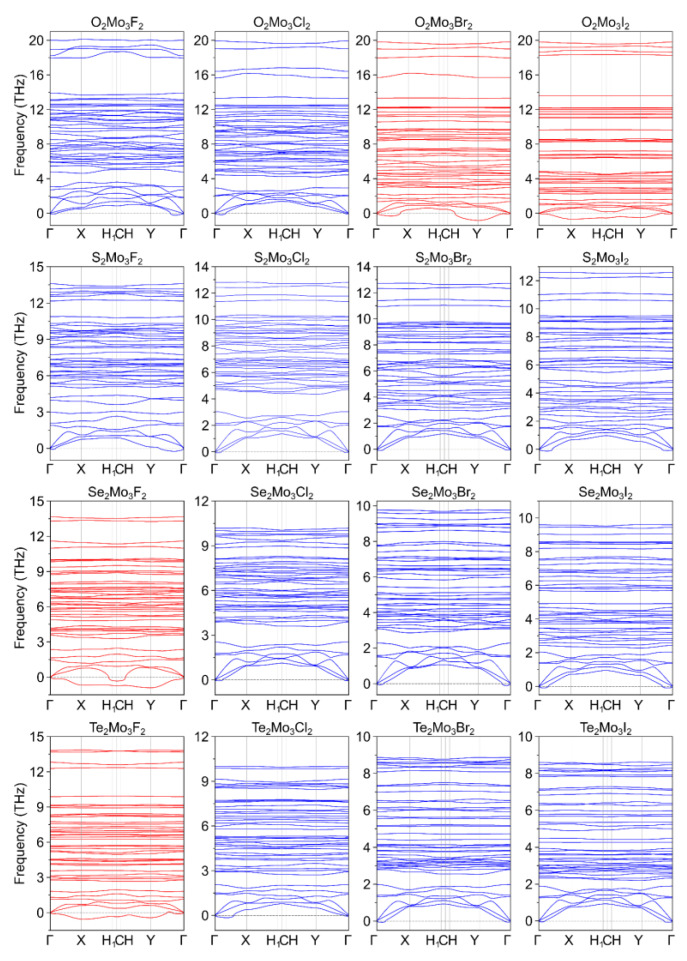
Phonon dispersion spectra for Ch_2_Mo_3_Hal_2_ (Ch = O, S, Se, Te and Hal = F, Cl, Br, I) Chevrel phases.

The dynamic stability of the two classes of Janus Chevrel monolayers—Ch^1^_2_Mo_3_Ch^2^_2_ (Ch^1^/Ch^2^ = O, S, Se, Te) and Ch_2_Mo_3_Hal_2_ (Ch = O, S, Se, Te and Hal = F, Cl, Br, I)—was investigated through phonon dispersion spectrum calculation. The small bulges in the vicinity of the Γ-point in the phonon dispersion spectra are known to be attributed to the lack of consideration of rotational invariance [71,72] and, therefore, not indicate the instability of the structure (see Figure 2 and Figure 3). According to the performed analysis, we can identify that only Se_2_Mo_3_O_2_ and Te_2_Mo_3_O_2_ among 2D Ch_2_Mo_3_Ch_2_ Janus structures and O_2_Mo_3_Br_2_, O_2_Mo_3_I_2_, Se_2_Mo_3_F_2_, and Te_2_Mo_3_F_2_ monolayers among 2D Ch_2_Mo_3_Hal_2_ Janus structures fail to exhibit dynamic stability as indicated by negative (below zero) phonon dispersive energy in at least one of the X, Y, H_1_, C, and H points of the Brillouin zone.

**Figure 4:**
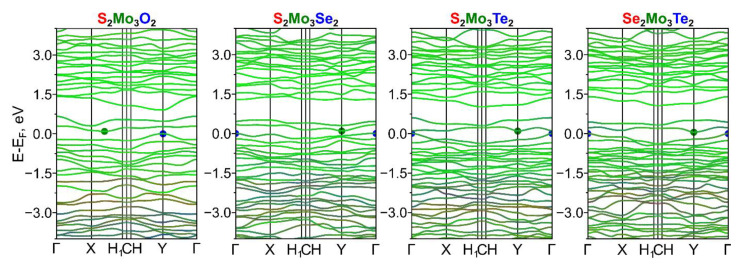
Atom-resolved electronic band structure of dynamically stable Janus Ch^1^_2_Mo_3_Ch^2^_2_ (Ch^1^/Ch^2^ = O, S, Se, Te) Chevrel phases. The contributions from Ch^1^, Ch^2^_,_ and Mo atoms are indicated with red, blue, and green colors, respectively.

**Figure 5:**
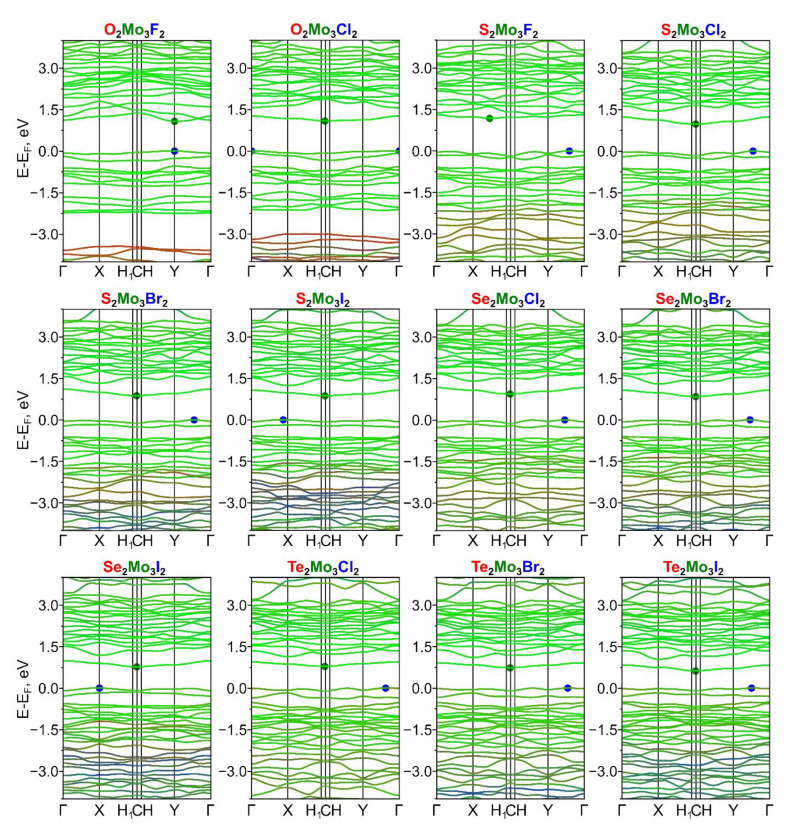
Atom-resolved electronic band structure of dynamically stable Janus Ch_2_Mo_3_Hal_2_ (Ch = O, S, Se, Te and Hal = F, Cl, Br, I) Chevrel phases. The contributions from Ch, Hal_,_ and Mo atoms are indicated with red, blue, and green colors, respectively.

We investigated the electronic properties of dynamically stable Ch^1^_2_Mo_3_Ch^2^_2_ (Ch^1^/Ch^2^ = O, S, Se, Te) and Ch_2_Mo_3_Hal_2_ (Ch = O, S, Se, Te and Hal = F, Cl, Br, I) monolayers. Non-Janus Mo_3_S_4_ Chevrel monolayers exhibit semiconducting properties with a band gap value of ~0.2 eV, while Mo_3_Te_4_ monolayers are metals (see Figure S2). Electronic band structures for the considered Janus monolayers are presented in Figure 4 and Figure 5. The substitution of chalcogen atoms on one side of the Chevrel non-Janus monolayer leads to the opening/increase of the band gap, and all considered monolayers exhibit semiconducting electronic properties (see Table 3). The halogen atoms’ influence on the band gap increasing is more significant than chalcogen and the band gap values range from near-zero values in the case of Se_2_Mo_3_Te_2_ to 1.17 eV in the case of S_2_Mo_3_F_2_. All Janus Chevrel monolayers except for O_2_Mo_3_F_2_ have an indirect band gap, while O_2_Mo_3_F_2_ is a direct band gap semiconductor. In all cases, the main contribution to the valence band maximum (VBM) and conduction band minima (CBM) comes from the molybdenum atoms. The features of the monolayer type of the Chevrel phase are that the molybdenum atoms are in a square pyramidal environment making their *d*-orbitals available for interaction with adsorbate molecules, which is useful for catalytic applications. In this case, the replacement of chalcogen atoms by halogen atoms leads to electron density redistribution among the surface Mo atoms since the electronegativity difference makes these Mo atoms attractive to ion sorption. Bader charge analysis of surface Mo atoms (see Table S1) shows similar values of electron deficiency on Mo atoms, which depend on the chalcogen or halogen type and do not depend on its combination in the Janus structure. For example, the Mo atom on the S side has an electron deficiency of 0.89 *e*, which makes this atom attractive for negatively charged ions.

To be applied in water-splitting reactions, the CBM of the non-Janus monolayer must be higher than the reduction level of hydrogen, while the VBM must be lower than the oxidation level of oxygen [73], resulting in restriction of the band gap value: E_g_ > 1.23 eV [73]. The feature of Janus monolayers is the presence of two inequivalent surfaces—(001) and $\left(00\bar{1}\right)$—leading to the appearance of an intrinsic dipole moment, resulting in the electrostatic potential difference between the opposite sides of the monolayers. The intrinsic dipole moment in the Janus monolayer leads to the modification of the restriction on the band gap value:

E_g_ > 1.23 − ΔΦ (1)

where ΔΦ is the difference between the vacuum levels of the opposite sides of Janus monolayers. Additionally, the necessary condition of the photocatalytic water-splitting process is the following: the VBM of the (001) surface must be lower than the oxidation potential, while the CBM of the $\left(00\bar{1}\right)$ surface must be higher than the reduction potential [73]. It should be noted that in actuality, the redox potentials of water are pH resistant [74].

**Figure 6:**
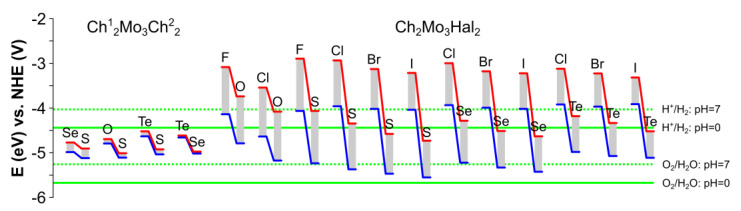
Band edge positions of dynamically stable Janus Ch^1^_2_Mo_3_Ch^2^_2_and Ch_2_Mo_3_Hal_2_ Chevrel monolayer (Ch^1^/Ch^2^ = O, S, Se, Te; Hal = F, Cl, Br, I) structures compared with the redox potentials of water. The values are given concerning the vacuum level (in eV). The CBM and VBM are marked by red and blue lines. The redox potentials of water are denoted as the green lines for pH = 7 (dashed) and pH = 0 (solid) [74].

The band edge positions of dynamically stable Ch^1^_2_Mo_3_Ch^2^_2_ (Ch^1^/Ch^2^ = O, S, Se, Te) and Ch_2_Mo_3_Hal_2_ (Ch = O, S, Se, Te and Hal = F, Cl, Br, I) monolayers, taking into account the influence of the intrinsic dipole moment concerning the redox potentials of water at pH = 0 and pH = 7, are presented in Figure 6. According to the obtained results, Ch^1^_2_Mo_3_Ch^2^_2_ Janus monolayers do not provide either the reaction of hydrogen production or the reaction of oxygen generation at both considered values of pH; therefore, these structures are not consistent for photocatalytic water splitting.

Most of the Ch_2_Mo_3_Hal_2_ monolayers belong to the type-1 material in neutral and acidic media, according to [75], as this material is suitable for only the HER or the OER due to the band alignments (see Table S2).

Overall water splitting can be achieved for S_2_Mo_3_I_2_ in neutral media and S_2_Mo_3_Br_2_ in alkali media. A possible way to tune electronic properties is through a variation of halogen content on one side of the Janus monolayer. The replacement of a Br with an S atom (S_2_Mo_3_Br_2_) leads to CBM shifting on the halogen side, which makes this material suitable for HER in neutral and acidic media (see Figure S3). However, the reverse side of the coin is that CBM will shift upwards on the chalcogen side.

One more important property of the catalyst material in the case of the photocatalytic process is the ability of the material to absorb the energy of incident solar radiation. To characterize the considered materials, we calculated the optical properties by considering the complex dielectric function $\varepsilon \left(\lambda \right)=\varepsilon_{1}\left(\lambda \right)+i\varepsilon_{2}\left(\lambda \right)$, in which the real part $\varepsilon_{1}\left(\lambda \right)$ was calculated using the Kramers–Kronig relation, while the imaginary part $\varepsilon_{2}\left(\lambda \right)$ was determined by the sum of empty states [76,77]. The wavelength-dependent extinction coefficient in the perpendicular direction and parallel to the surface of the monolayer was calculated according to [78] as:

(2) $$K\left(\lambda \right)={\left[\frac{\sqrt{\varepsilon_{1}^{2}\left(\lambda \right)+\varepsilon_{2}^{2}\left(\lambda \right)}-\varepsilon_{1}\left(\lambda \right)}{2}\right]}^{\frac{1}{2}}$$

**Figure 7:**
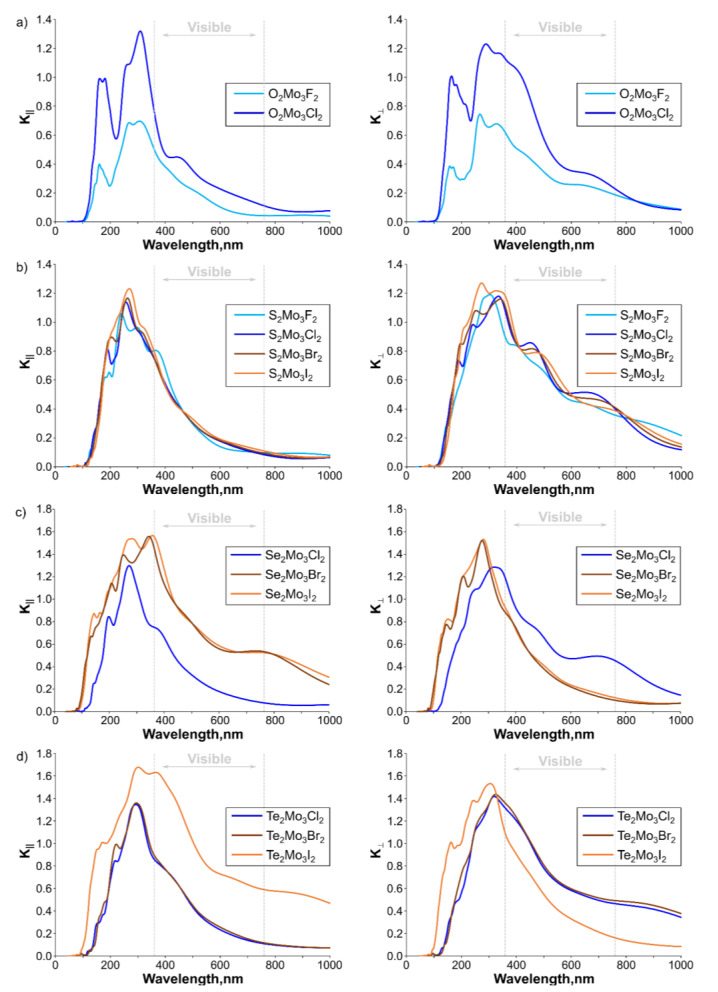
Wavelength dependence of the extinction coefficient in the transverse direction and perpendicular to the surface for dynamically stable Janus Ch_2_Mo_3_Hal_2_ Chevrel phases (Ch = O, S, Se, Te; Hal = F, Cl, Br, I): (**a**) oxygen-, (**b**) sulfur-, (**c**) selenium-, and (**d**) tellurium-containing monolayers.

The resulting dependences for the Janus monolayers suitable for a catalytic process are presented in Figure 7, whileCh^1^_2_Mo_3_Ch^2^_2_ and Mo_3_Ch are presented_4_ in Figures S4 and S5, respectively. Overall, Janus Chevrel monolayers suitable for catalysis (Figure 7) are characterized by a strong peak at 250–300 nm in both the transverse direction and perpendicular to the surface, which correspond to the UV region. In the case of oxygen-containing Janus structures, there is also a separate peak at 150–160 nm (UV region) in both the transverse direction and perpendicular to the surface. In the case of Te-containing monolayers, the intensity of the extinction coefficient decreases compared with pristine monolayers (Figure S4), while in other cases, the intensity is comparable. Overall, Janus monolayers can absorb the light at ultraviolet and visible spectral ranges, indicating a high potential for application in photocatalytic water-splitting reactions and as an element of optoelectronic devices.

**Figure 8:**
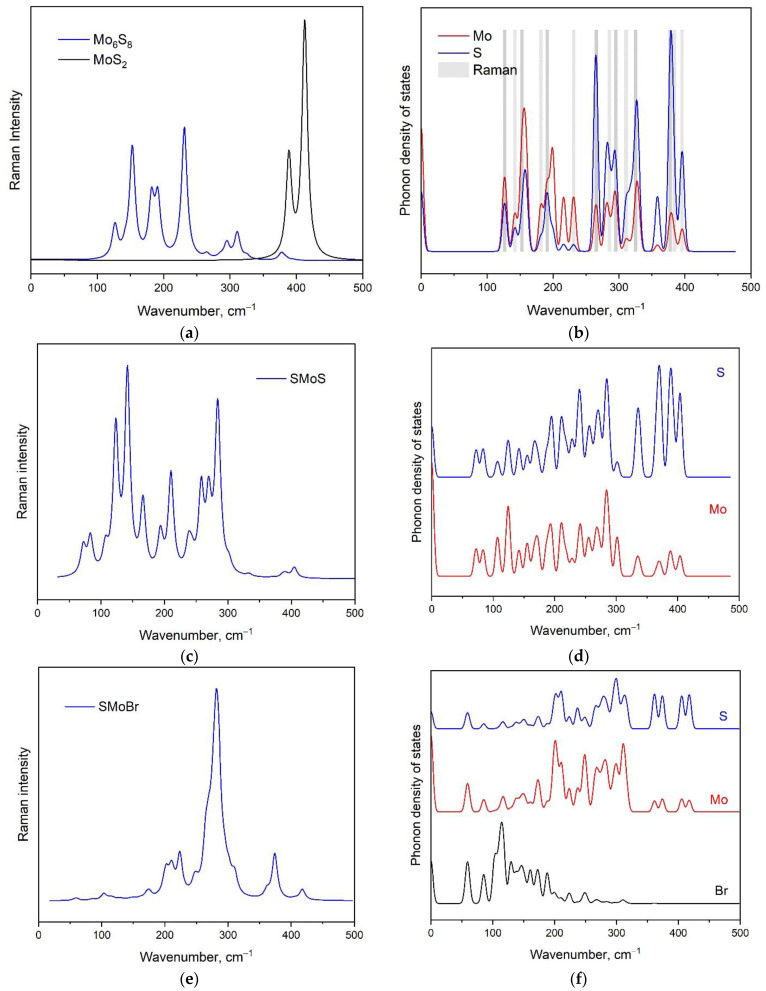
Simulated Raman spectra of the Chevrel phase of (**a**) bulk Mo_6_S_8_ (blue) and MoS_2_ (black); (**c**) Mo_3_S_4_ monolayer; (**e**) S_2_Mo_3_Br_2_ monolayer. The corresponding phonon densities of states are shown in (**b**,**d**,**f**), respectively.

To facilitate the experimental identification of Chevrel monolayers, we simulated vibrational spectra for the initial bulk Mo_6_S_8_ Chevrel crystal [79] (Figure 8a,b) and the individual Mo_6_S_8_ monolayer (Figure 8c,d). Experimental Raman spectra of bulk Mo_6_S_8_ found in [21,80] indicate the existence of two strong sharp peaks in a high-wavenumber region associated with Mo–S stretching vibrations [21]. The same strong peaks were observed in the case of NiMo_3_S_4_ [81]; however, no such peaks were found in the case of BaMo_6_S_8_, Cu_1.8_Mo_6_S_8_, Cu_3.2_Mo_6_S_8_, PbMo_6_S_8_, and SnMo_6_S_8_ crystals with a Chevrel structure [82]. In [83], the Raman spectra of Mo_6_S_3_I_6_ nanowires are presented in a wide range of temperatures and the existence of such peaks is shown in the case of samples synthesized at 900 and 1000 °C. At temperatures above 1000 °C, a powder XRD pattern of Mo_6_S_3_I_6_ no longer contains lines fitting with MoS_2_ and the related bands disappear from the Raman spectra. In Figure 8a, it can be seen that the high-wavenumber region of the simulated spectrum of bulk Mo_6_S_8_ does not contain these strong bands, but if we consider the combination of bulk Mo_6_S_8_ and bulk MoS_2_ spectra, the resulting figure (Figure 8a) is in good agreement with the experimental data from [21,80]. Thus, we suppose that the samples studied in [21,80,81] can have the impurities of MoS_2_. Due to the complexity of the Mo_6_S_8_ structure, the Raman spectrum obtained in this work theoretically can be assigned based on the phonon density of states calculation (see Figure 8b). The contribution of sulfur ion vibrations is significantly above 250 cm^−1^. A medium band at 181 cm^−1^ and a strong band at 231 cm^−1^ are associated with Mo translation. The low-wavenumber spectral range contains both Mo and S ion vibrations.

The phonon DOS for the Mo_3_S_4_ Chevrel monolayer is shown in Figure 8d. Despite the large sulfur phonon density of states in the high-wavenumber region (above 300 cm^−1^), the Raman intensities in the spectrum shown in Figure 8d are weak. There are no totally symmetric vibrations of the Mo_3_S_4_ Chevrel monolayer like in the case of MoS_2_ [84]. For example, the vibrational mode with the highest wavenumber value (404 cm^−1^) involves only half of the sulfur ions on each monolayer surface and, at the same time, the sulfur ions remain motionless on the opposite surface (see Figure S6a). Such behavior is similar to the MoSSe Janus monolayers [85]. In this case, the vibration of only one side of the monolayer does not manifest itself as a strong, medium, or even weak Raman band. The strong band at 284 cm^−1^ is associated with in-plane ions’ movement, as shown in Figure S6b. The strong spectral band at 210 cm^−1^ can be described as the stretching of a Mo_3_S_4_ Chevrel-like molecule, as presented in Figure S6c. The strong lines at 141 cm^−1^ (Figure S6d) and 123 cm^−1^ are out-of-plane and in-plane vibrations, respectively. Meanwhile, the spectral lines in Figure 8c above 100 cm^−1^ can be described as the translations of ions, and the contribution of these ions can be determined using Figure 8d. Raman bands below 100 cm^−1^ are assigned as the rotations of Mo_3_S_4_ Chevrel-like molecules (see Figure S6c).

As a representative of Ch_2_Mo_3_Hal_2_ Chevrel monolayers, we considered the S_2_Mo_3_Br_2_ monolayer and simulated its Raman spectra as dynamically stable and suitable for the photocatalytic process (Figure 8e). According to the phonon density of states shown in Figure 8f, the vibrations of S and Mo ions contribute to the main intense spectral bands of the S_2_Mo_3_Br_2_ Raman spectrum (from 200 to 417 cm^−1^). Bromine vibrations are in the region below 200 cm^−1^; however, the calculated intensities of corresponding Raman bands are weak. The low-intensity bands above 350 ^−1^cm in the Raman spectrum of S_2_Mo_3_Br_2_ are mainly associated with S vibrations, the forms of which are shown in Figure S6f,g. The strongest spectral band in Figure 8e is associated with Mo–S stretching vibrations. The group of spectral bands from 200 to 235 cm^−1^ can be attributed to antisymmetric movements of S ions as, for example, are shown in Figure S6h.

## 4. Conclusions

The photocatalytic water-splitting materials can most likely be found in Janus-type structures consisting of transition metals, chalcogen, and halogen atoms. Here, we theoretically investigated a novel type of Janus structure with the Chevrel phase topology and considered the physical properties and stability. The calculated exfoliation energy from the corresponding bulk chalcogenides demonstrated the possibility of monolayer exfoliation, which can be transformed to suit Janus structures by replacing chalcogen atoms with halogen atoms from one side of the monolayer. The electronic structure calculations demonstrated that halogen doping increases the band gap compared with non-Janus structures, which makes these materials appropriate for the HER and the OER. Among all the considered 2D structures, S_2_Mo_3_I_2_ is the most promising candidate for overall water splitting due to the appropriate band alignments relative to the redox potentials of water and the ability to absorb at the UV and visible regions of the light spectra. The calculated Raman spectra can play the role of characteristic blueprints, facilitating the experimental identification and verification of the considered structures. The theoretical results that were obtained open up the prospects for the experimental synthesis and investigation of new compounds based on the Chevrel monolayer phase for photocatalytic as well as for other technological applications.

## Figures and Tables

**Table 1:** Unit cell parameters for Ch_2_Mo_3_Ch_2_ (Ch = S, Se, Te) Chevrel monolayers.

Ch^1^_2_Mo_3_Ch^2^_2_ Monolayer	a, Å	b, Å	γ, °
S_2_Mo_3_S_2_	6.518	6.623	84.89
Se_2_Mo_3_Se_2_	6.644	6.738	85.10
Te_2_Mo_3_Te_2_	6.834	6.902	89.73
S_2_Mo_3_Se_2_	6.644	6.738	85.10
S_2_Mo_3_O_2_	6.190	6.425	84.10
S_2_Mo_3_Te_2_	6.834	6.902	89.73
Se_2_Mo_3_O_2_	6.499	6.543	88.66
Se_2_Mo_3_Te_2_	6.943	6.957	87.39
Te_2_Mo_3_O_2_	6.861	7.074	90.92

**Table 2:** Unit cell parameters for Ch_2_Mo_3_Hal_2_ (Ch = O, S, Se, Te; Hal = F, Cl, Br, I) Chevrel monolayers.

Ch_2_Mo_3_Hal_2_ Monolayer	a, Å	b, Å	γ, °
O_2_Mo_3_F_2_	6.148	6.131	85.87
O_2_Mo_3_Cl_2_	6.362	6.286	87.38
O_2_Mo_3_Br_2_	6.864	6.436	87.31
O_2_Mo_3_I_2_	7.337	7.328	84.04
S_2_Mo_3_F_2_	6.517	6.521	86.01
S_2_Mo_3_Cl_2_	6.656	6.637	86.49
S_2_Mo_3_Br_2_	6.791	6.773	86.60
S_2_Mo_3_I_2_	7.061	7.033	86.38
Se_2_Mo_3_F_2_	6.720	6.741	85.78
Se_2_Mo_3_Cl_2_	6.796	6.780	86.45
Se_2_Mo_3_Br_2_	6.909	6.892	86.46
Se_2_Mo_3_I_2_	7.133	7.116	86.29
Te_2_Mo_3_F_2_	7.170	7.172	85.51
Te_2_Mo_3_Cl_2_	7.087	7.089	86.33
Te_2_Mo_3_Br_2_	7.142	7.135	86.34
Te_2_Mo_3_I_2_	7.291	7.277	86.17

**Table 3:** Band gap values (E_g_) and vacuum potential difference (ΔΦ) for opposite sides of dynamically stable Janus Ch_2_Mo_3_Hal_2_ and Ch^1^_2_Mo_3_Ch^2^_2_ (Ch^1^/Ch^2^ = O, S, Se, Te) Chevrel phases.

Monolayer	(001) Surface	$\left(00\bar{1}\right)$ Surface	E_g_, eV	ΔΦ, eV
S_2_Mo_3_Se_2_	Se	S	0.21	0.14
S_2_Mo_3_O_2_	O	S	0.10	0.32
S_2_Mo_3_Te_2_	Te	S	0.11	0.40
Se_2_Mo_3_Te_2_	Te	Se	0.05	0.36
O_2_Mo_3_F_2_	F	O	1.05	0.65
O_2_Mo_3_Cl_2_	O	Cl	1.10	0.54
S_2_Mo_3_F_2_	F	S	1.17	1.17
S_2_Mo_3_Cl_2_	Cl	S	1.03	1.41
S_2_Mo_3_Br_2_	Br	S	0.89	1.45
S_2_Mo_3_I_2_	I	S	0.82	1.51
Se_2_Mo_3_Cl_2_	Cl	Se	0.94	1.29
Se_2_Mo_3_Br_2_	Br	Se	0.82	1.33
Se_2_Mo_3_I_2_	I	Se	0.80	1.41
Te_2_Mo_3_Cl_2_	Cl	Te	0.80	1.06
Te_2_Mo_3_Br_2_	Br	Te	0.74	1.11
Te_2_Mo_3_I_2_	I	Te	0.59	1.20

## Data Availability

Data available on request.

## Supplementary Materials

The following supporting information can be downloaded at: https://www.mdpi.com/article/10.3390/nano13020368/s1, Table S1. Bader charge analysis for surface Mo atoms in Ch_2_Mo_3_Hal_2_ (Ch = O, S, Se, Te; Hal = F, Cl, Br, I) Chevrel monolayers; Table S2. Reaction types on Ch_2_Mo_3_Hal_2_ (Ch = O, S, Se, Te; Hal = F, Cl, Br, I) Chevrel monolayers; Figure S1. Phonon dispersion spectra for Ch_2_Mo_3_Ch_2_ (Ch = S, Se, Te) Chevrel monolayers; Figure S2. Electronic band structure for Ch_2_Mo_3_Ch_2_ (Ch = S, Se, Te) Chevrel monolayers; Figure S3. Band edge positions of Janus (Br_2_S_2_)Mo_6_S_4_, (Br_3_S)Mo_3_S_4_ and (Br_4_)Mo_3_S_4_ Chevrel monolayers compared with the redox potentials of water. The values are given concerning the vacuum level (in eV); Figure S4. Wavelength dependence of the extinction coefficient in the transverse direction and perpendicular to the surface for Janus Ch_2_Mo_3_Ch_2_ (Ch = S, Se, Te) Chevrel phases; Figure S5. Wavelength dependence of the extinction coefficient in the transverse direction and perpendicular to the surface for dynamically stable Janus Ch^1^_2_Mo_3_Ch^2^_2_ (Ch^1^/Ch^2^ = O, S, Se, Te) Chevrel phases; Figure S6. Vibrational modes in the Mo_3_S_4_ monolayer at (a) 404 cm^−1^, (b) 283 cm^−1^, (c) 210 cm^−1^, (d) 141 cm^−1^, and (e) 72 cm^−1^. Graphical presentation of vibrations in the S_2_Mo_3_Br_2_ monolayer at (f) 417 cm^−1^, (g) 374 cm^−1^, and (h) 209 cm^−1^.
