# Supplementary material for: Halogen-Doped Chevrel Phase Janus Monolayers for Photocatalytic Water Splitting
Source: Nanomaterials (Basel). 2023 Jan 16;13(2):368. doi: 10.3390/nano13020368 (PMC9860981; doi:10.3390/nano13020368)
Supplement: Supplementary file 1 [file nanomaterials-13-00368-s001.zip › nanomaterials-2163732-supplementary.pdf]

Supporting Information

for

Halogen-Doped Chevrel Phase Janus Monolayers for Photocatalytic Water Splitting

Ekaterina V. Sukhanova [1,*], Nursultan E. Sagatov [2], Aleksandr S. Oreshonkov [1,3,4], Pavel N. Gavryushkin [1,2,5] and Zakhar I. Popov [1,*]

1 Laboratory of Acoustic Microscopy, Emanuel Institute of Biochemical Physics of Russian Academy of Sciences, 119334 Moscow, Russia
2 Laboratory of Phase Transformations and State Diagrams of the Earth's Matter at High Pressures, Sobolev Institute of Geology and Mineralogy, Siberian Branch of Russian Academy of Sciences, prosp. acad. Koptyuga 3, 630090 Novosibirsk, Russia
3 Laboratory of Molecular Spectroscopy, Kirensky Institute of Physics, Federal Research Center KSC SB RAS, 660036 Krasnoyarsk, Russia
4 School of Engineering and Construction, Siberian Federal University, 660041 Krasnoyarsk, Russia
5 Geology geophysics department, Novosibirsk State University, Pirogova 2, 630090 Novosibirsk, Russia
* Correspondence: yekaterina.sukhanova@phystech.edu (E.V.S.); zipcool@bk.ru (Z.I.P.)

Table S1. Bader charge analysis for surface Mo atoms in $Ch_2Mo_3Hal_2$ (Ch = O, S, Se, Te; Hal = F, Cl, Br, I) Chevrel monolayers.

$Ch_2Mo_3Hal_2$ monolayer	Charge, *e*/Mo-atom	
	Chalcogen side	Halogen side
$O_2Mo_3F_2$	1.13	0.91
$O_2Mo_3Cl_2$	1.16	0.74
$S_2Mo_3F_2$	0.89	0.90
$S_2Mo_3Cl_2$	0.89	0.72
$S_2Mo_3Br_2$	0.89	0.60
$S_2Mo_3I_2$	0.89	0.45
$Se_2Mo_3Cl_2$	0.74	0.72
$Se_2Mo_3Br_2$	0.74	0.59
$Se_2Mo_3I_2$	0.74	0.45
$Te_2Mo_3Cl_2$	0.52	0.73
$Te_2Mo_3Br_2$	0.52	0.61
$Te_2Mo_3I_2$	0.52	0.45

Table S2. Reaction types on $Ch_2Mo_3Hal_2$ (Ch = O, S, Se, Te; Hal = F, Cl, Br, I) Chevrel monolayers.

$Ch_2Mo_3Hal_2$ monolayer	Reaction type	
	Chalcogen side	Halogen side
$O_2Mo_3F_2$	HER	HER
$O_2Mo_3Cl_2$	HER	HER
$S_2Mo_3F_2$	HER	HER
$S_2Mo_3Cl_2$	HER/OER	-
$S_2Mo_3Br_2$	OER	-
$S_2Mo_3I_2$	OER	HER
$Se_2Mo_3Cl_2$	HER	-
$Se_2Mo_3Br_2$	OER	-
$Se_2Mo_3I_2$	OER	-
$Te_2Mo_3Cl_2$	HER	-
$Te_2Mo_3Br_2$	HER	-
$Te_2Mo_3I_2$	-	-

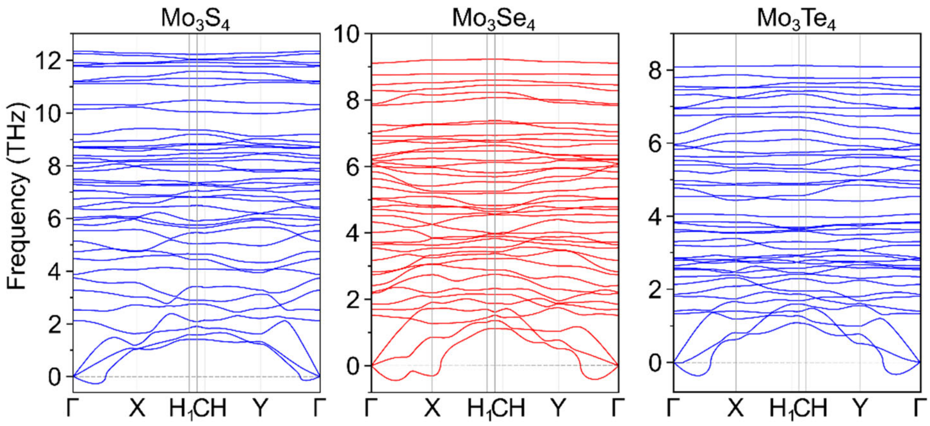

Figure S1. Phonon dispersion spectra for $Ch_2Mo_3Ch_2$ (Ch = S, Se, Te) Chevrel monolayers.

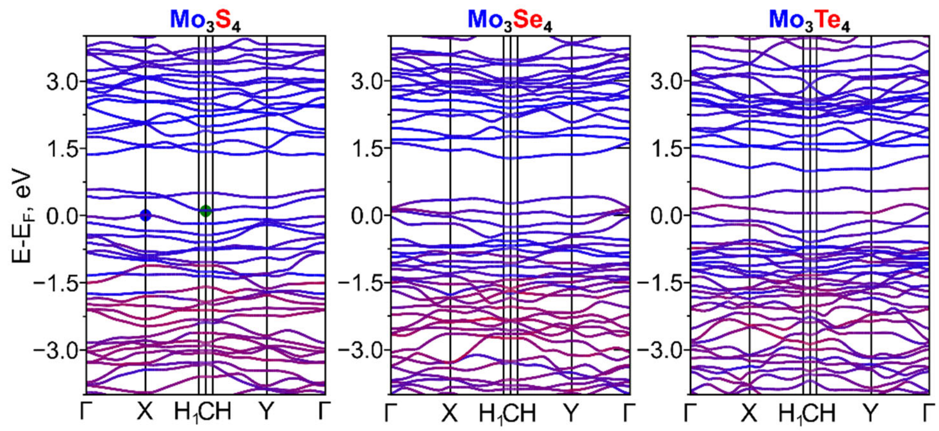

Figure S2. Electronic band structure for $Ch_2Mo_3Ch_2$ (Ch = S, Se, Te) Chevrel monolayers.

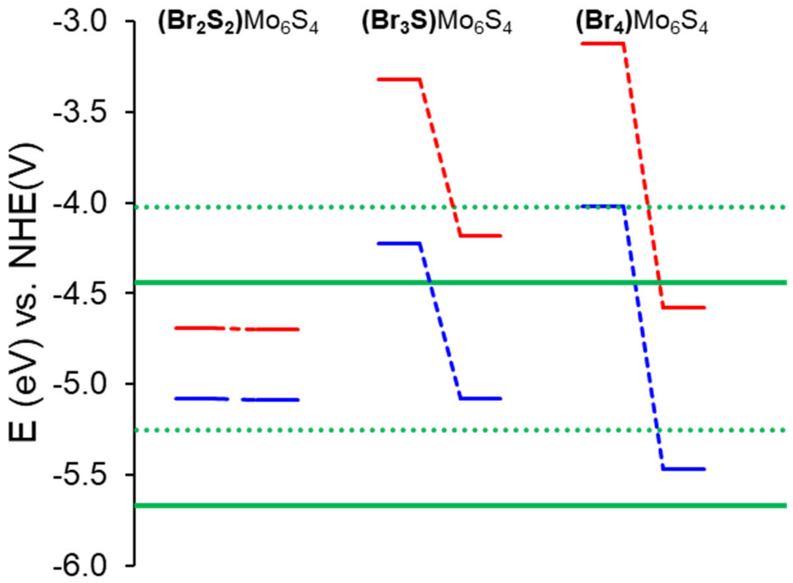

Figure S3. Band edge positions of Janus $(Br_2S_2)Mo_6S_4$, $(Br_3S)Mo_3S_4$ and $(Br_4)Mo_3S_4$ Chevrel monolayers compared with the redox potentials of water. The CBM and VBM are marked by red and blue lines. The values are given concerning the vacuum level (in eV). The redox potentials of water are denoted as green lines for pH=7 (dashed) and pH=0 (solid) [1].

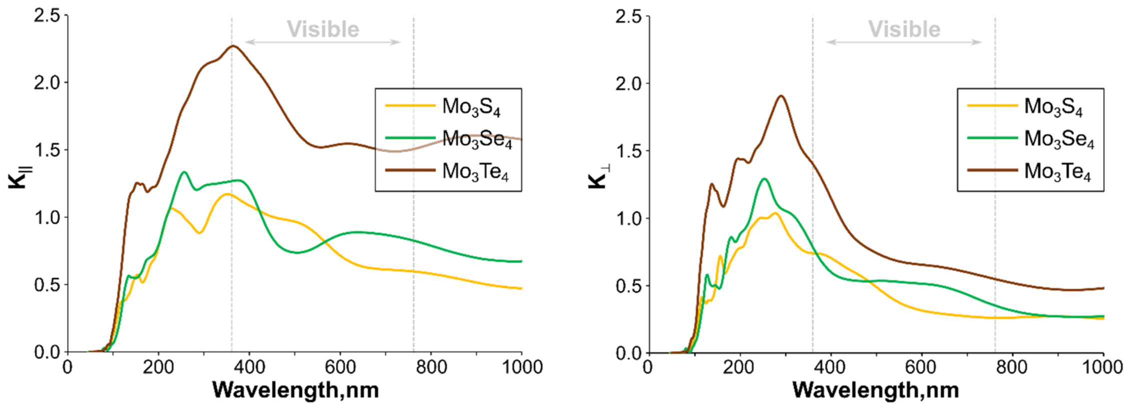

Figure S4. Wavelength dependence of the extinction coefficient in the transverse and perpendicular to the surface direction for Janus $Ch_2Mo_3Ch_2$ (Ch = S, Se, Te) Chevrel phases.

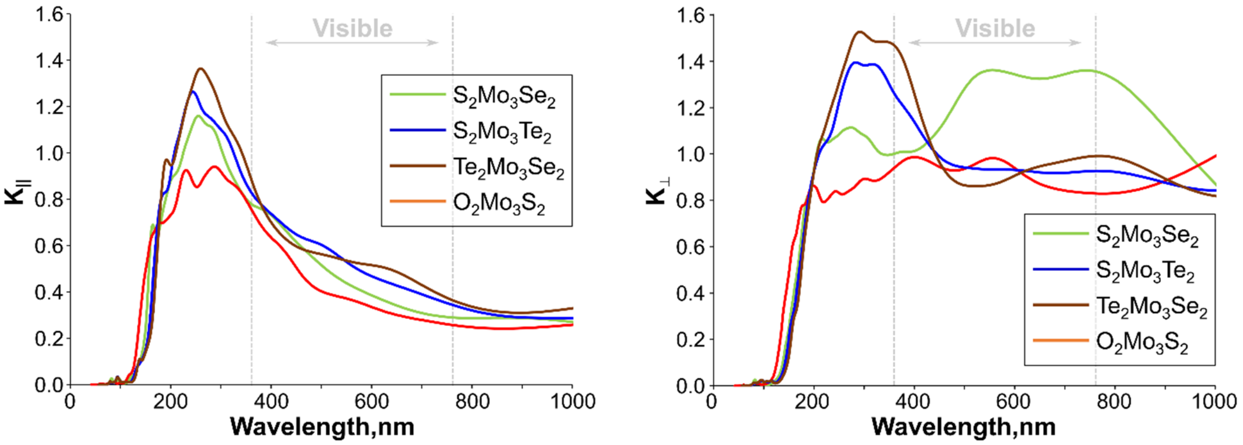

Figure S5. Wavelength dependence of the extinction coefficient in the transverse and perpendicular to the surface direction for dynamically stable Janus $Ch^1{}_2Mo_3Ch^2{}_2$ (Ch^1/Ch^2 = O, S, Se, Te) Chevrel phases.

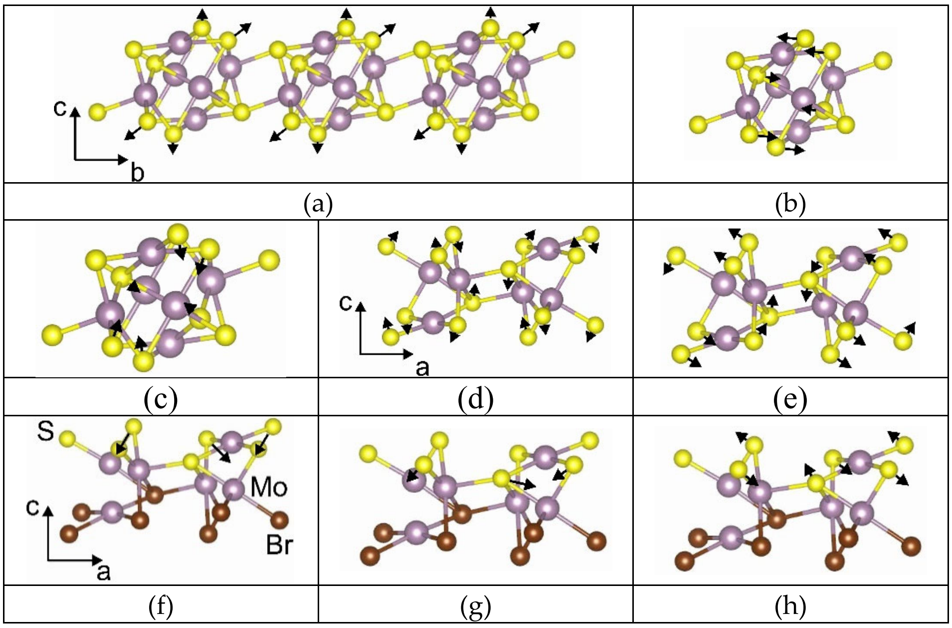

Figure S6. Vibrational modes in Mo_3S_4 monolayer at (a) 404 cm^{-1}, (b) 283 cm^{-1}, (c) 210 cm^{-1}, (d) 141 cm^{-1}, (e) 72 cm^{-1}. Graphical presentation of vibrations in $S_2Mo_3Br_2$ monolayer at (f) 417 cm^{-1}, (g) 374 cm^{-1}, (h) 209 cm^{-1}.

References

1. Chakrapani, V.; Angus, J.C.; Anderson, A.B.; Wolter, S.D.; Stoner, B.R.; Sumanasekera, G.U. Charge Transfer Equilibria between Diamond and an Aqueous Oxygen Electrochemical Redox Couple. *Science* **2007**, *318*, 1424–1430, doi:https://doi.org/10.1021/acs.nanolett.8b02561.
